# Orange/Red Photoluminescence Enhancement Upon SF_6_ Plasma Treatment of Vertically Aligned ZnO Nanorods

**DOI:** 10.3390/nano9050794

**Published:** 2019-05-23

**Authors:** Amine Achour, Mohammad Islam, Sorin Vizireanu, Iftikhar Ahmad, Muhammad Aftab Akram, Khalid Saeed, Gheorghe Dinescu, Jean-Jacques Pireaux

**Affiliations:** 1 Laboratoire Interdisciplinaire de Spectroscopie Electronique (LISE), Namur Institute of Structured Matter (NISM), University of Namur, 61 Rue de Bruxelles, 5000 Namur, Belgium; a_aminph@yahoo.fr (A.A.); jean-jacques.pireaux@unamur.be (J.-J.P.); 2 Center of Excellence for Research in Engineering Materials, Deanship of Scientific Research, King Saud University, P.O. Box 800, Riyadh 11421, Saudi Arabia; ifahmad@ksu.edu.sa; 3 National Institute for Laser, Plasma and Radiation Physics, Magurele, P.O. Box MG-16, 077125 Bucharest, Romania; s_vizi@infim.ro (S.V.); dinescug@infim.ro (G.D.); 4 School of Chemical and Materials Engineering, National University of Sciences and Technology, Sector H-12, Islamabad 44000, Pakistan; aftabakram@scme.nust.edu.pk; 5 Department of Mechanical Engineering, College of Engineering, King Saud University, P.O. Box 800, Riyadh 11421, Saudi Arabia; khaliduetp@gmail.com

**Keywords:** ZnO nanorods, photoluminescence, SF_6_ plasma, visible emission, red emission

## Abstract

Although the origin and possible mechanisms for green and yellow emission from different zinc oxide (ZnO) forms have been extensively investigated, the same for red/orange PL emission from ZnO nanorods (nR) remains largely unaddressed. In this work, vertically aligned zinc oxide nanorods arrays (ZnO nR) were produced using hydrothermal process followed by plasma treatment in argon/sulfur hexafluoride (Ar/SF_6_) gas mixture for different time. The annealed samples were highly crystalline with ~45 nm crystallite size, (002) preferred orientation, and a relatively low strain value of 1.45 × 10^−3^, as determined from X-ray diffraction pattern. As compared to as-deposited ZnO nR, the plasma treatment under certain conditions demonstrated enhancement in the room temperature photoluminescence (PL) emission intensity, in the visible orange/red spectral regime, by a factor of 2. The PL intensity enhancement induced by SF_6_ plasma treatment may be attributed to surface chemistry modification as confirmed by X-ray photoelectron spectroscopy (XPS) studies. Several factors including presence of hydroxyl group on the ZnO surface, increased oxygen level in the ZnO lattice (*O_L_*), generation of F–OH and F–Zn bonds and passivation of surface states and bulk defects are considered to be active towards red/orange emission in the PL spectrum. The PL spectra were deconvoluted into component Gaussian sub-peaks representing transitions from conduction-band minimum (CBM) to oxygen interstitials (*O_i_*) and CBM to oxygen vacancies (*V_O_*) with corresponding photon energies of 2.21 and 1.90 eV, respectively. The optimum plasma treatment route for ZnO nanostructures with resulting enhancement in the PL emission offers strong potential for photonic applications such as visible wavelength phosphors.

## 1. Introduction

With a direct band gap and high exciton binding energy values of 3.3 eV and 60 meV, respectively, the transparent n-type semiconductor zinc oxide (ZnO) has demonstrated use for numerous electronic, electrochemical, optoelectronic, and electromechanical devices [1,2,3,4], including light-emitting diodes [5], piezoelectric nanogenerators [6], field emission devices [7], high-performance nanosensors [8,9], solar cells [10,11], and ultraviolet (UV) lasers [12]. Due to their high surface-to-volume ratio and surface area, the ZnO nanostructures with zero- (quantum dots and ultrafine nanoparticles), one- (tubes, rods, belts, and wires), or two-dimensional morphology (sheets, flakes, and thin films) offer superior performance attributes than those of bulk ZnO structures. 

The enhancement of visible emission from ZnO is also important besides any improvement in its UV emission characteristics. The presence of intrinsic defects in the ZnO lattice structure causes photoemission in the green or yellow/orange/red spectral regime. The visible photoluminescence (PL) emission in ZnO quantum dots (QD) was found to depend on the QD size as well as presence of singly ionized oxygen vacancies as defects and was ascribed to the transition of holes from valence band to preexisting deep donor energy levels [13]. Another study reported the effect of evaporation or chemical methods, as processing route, on the extent of green or yellow emission due to surface centers or defects in the bulk ZnO [14]. A decrease in aspect ratio owing to a change from nanoneedle to nanorod morphology was found to induce an increase in the PL intensity via nonradiative quenching by near-surface defects [15]. Various approaches explored for any enhancement in the visible PL emission include (i) control and induction of abundant intrinsic point defects such as zinc interstitials (*Zn_i_*) and oxygen vacancies (*V_O_*) in the ZnO nanostructures [16], (ii) doping with rare-earth ions [17,18], (iii) ZnO annealing and exposure to charge scavengers [19], and (iv) surface plasmon effect due to coupling with Au or Ag nanoparticles [20,21]. 

The solution processing routes offer an outstanding merit in terms of low equipment infrastructure and manufacturing costs. Most of these processes are also amenable to optoelectronic device fabrication that require vertical stacking of heterostructures. In this context, hydrothermal synthesis carries certain advantages, namely low processing temperatures, and inherent flexibility to in situ fabrication and integration. For intrinsic, doped, and core/shell ZnO nanorods arrays, hydrothermal process is a promising technique for tunable composition and desirable optical properties. In addition to other parameters, the shape and morphology of the resulting nanorods strongly depend on the ZnO source molarity in the solution, with hexagonal, faceted growth at high concentrations [18]. 

An investigation into the film thickness (70–220 nm) and annealing temperature (200–900 °C) effects on the atomic layer deposition ZnO films revealed green, orange, or red emission due to transition of the photoexcited electrons from the *CB* (conduction band) (2.17 eV) to *V_O_*, *Zn_i_* to *O_i_* (interstitial oxygen) (2.09 eV), or from the *CB* to the mid-gap states (1.79–1.99 eV), respectively [22]. The lattice disorder or structural imperfections such as grain boundaries and surface defects in the ZnO crystals give rise to *O_i_* and *O_Zn_* oxygen antisites, thus causing orange/red emission [23]. Various mechanisms have been proposed for green emission based on *V_Zn_*, *V_O_*, *Zn_i_*, and *O_i_*, or *O_Zn_* and *O_i_* antisites [23,24,25,26,27]. Other mechanisms that may be active involve substituted Cu atoms at significantly high concentration, shallow donor to deep acceptor electronic transitions [25]. 

The PL visible emission from ZnO nanostructures can be tailored through surface modification treatments such as hydrogenation, polymer covering, argon ion milling, and annealing in different ambiences [28,29]. Polydorou et al. [30] reported an increase in the near band edge (NBE) intensity and reduction in the broad band intensity in the visible range upon ZnO thin film treatment in pure and mixed SF_6_ plasma with subsequent improvements in device efficiency and lifetime of inverted polymer solar cells. On the other hand, Prucnal et al. [31] noticed an increase in the PL intensities for both UV (near-band-edge or NBE) and visible range (deep level emission or DLE) upon SF_6_ plasma treatment of the ZnO films for 125 s. Treatment of the ZnO nanorods with hydrogen or oxygen plasma has been found to reduce *V_O_* concentration and increase *O_i_* concentration, respectively, with subsequent intensity changes in the PL spectra [32]. Yet, another study of the Ar plasma treatment of the ZnO nanowires revealed intensity enhancement of the NBE emission over DLE [33]. Although there are several reports on processing or plasma-induced surface modification of ZnO thin films and nanostructures, the relative enhancement in the visible emission (DLE) still needs to be further explored.

In this paper, we performed in-depth PL and XPS investigations of the as-produced and after argon/sulfur hexafluoride (Ar/SF_6_) plasma treatment of ZnO nanorods (ZnO nR) produced by hydrothermal technique. The ZnO nR plasma treatment was accomplished by means of low-pressure plasmas generated by electrical discharges in Ar/SF_6_ gas mixtures. The plasma treatments under controlled conditions preserved the nano/microfeatures of the ZnO nR surface morphology, while incorporation of chemical groups was achieved [34]. The surface chemistry of the samples was assessed by X-ray photoelectron spectroscopy which is a powerful surface characterization technique allowing detailed investigation of the chemical bonding. As compared to the initial ZnO nR, the photoluminescence (PL) emission in the visible region was doubled after the plasma treatment.

## 2. Experimental Procedure

### 2.1. Synthesis of ZnO Nanorods

10 mM zinc acetate dihydrate (ZAD, Zn(CH_3_COO)_2_·2H_2_O) (98% purity, Sigma Aldrich, St. Louis, MO, USA) was dissolved in ethanol and the solution was spin-coated onto indium tin oxide (ITO) coated glass substrates at a 2000 rpm for 30 s. The spin casting cycle was repeated 3–5 times, and after each deposition cycle the samples were air dried at 130 °C for 5 min. Finally, a seed layer of ZnO nanoparticles was produced by annealing the samples in air at 340 °C for 10 min. Over the seeded substrates, ZnO nanorods were grown through immersion in 25 mM zinc nitrate hexahydrate (Zn(NO_3_)_2_·6H_2_O) and 25 mM hexamethylenetetramine ((CH_2_)_6_N_4_, HMTA) aqueous solution mixture at 90 °C for 6 h in a sealed autoclave. At high temperature in the oven, HMTA not only provides the basic environment needed for Zn(OH)_2_ formation, it also performs coordination with and stabilization of Zn^2+^ ions, and subsequent dehydration into ZnO [35]. After that, the samples were extracted from the autoclave, washed thoroughly several times with deionized water to remove any residual reactants, and dried in air. As a final setup, the samples were annealed in air at 350 °C for 10 min to get rid of any trace of the organic precursor, as described elsewhere [36]. 

### 2.2. Ar/SF_6_ Plasma Treatment

The as-produced ZnO nanorods were treated with a mixture of argon (Ar) and sulfur hexafluoride (SF_6_) plasma using a glass bell jar reactor [37]. A capacitively coupled radio-frequency discharge (RF, 13.56 MHz) was generated between two parallel planar electrodes that were 4 cm apart. The upper electrode was connected to the RF power supply whereas the lower, grounded electrode was used as the substrate holder. The Ar/SF_6_ gas mixture with volumetric flow rates of 10 and 20 sccm for the Ar and SF_6_ gases, respectively, was introduced into the chamber. The plasma treatment was performed at 25 W applied RF power and 15 Pa working pressure for different time durations. Throughout this work, the as-prepared and plasma-treated zinc oxide nanorods arrays (after 5, 10, and 20 min of Ar/SF_6_ plasma treatment) are referred to as Z-nR, Z-nR5, Z-nR10, and Z-nR20, respectively. 

### 2.3. Nanostructures Characterization 

For characterizing their morphology and composition, the as-deposited ZnO nanorods samples were investigated using scanning electron microscope (SEM) and the X-ray diffraction (XRD) machine, respectively. The microstructural examination was carried out on a JSM7600 model (JEOL, Tokyo, Japan) operating at 5 kV. The XRD studies were performed on a D500 MOXTEK apparatus (MOXTEK, Inc., Orem, UT, USA) in the θ–2θ Bragg Brentano configuration with monochromatic Cu-K*α* radiation (λ = 1.5404 Å). The photoluminescence (PL) measurements were recorded from all samples at room temperature by means of a Jobin-Yvon Fluorolog^®^-3 spectrometer (Horiba Scientific, Piscataway, NJ, USA) with a 500 W Xenon lamp. To probe the surface composition of the nanostructures arrays, X-ray photoelectron spectroscopy (XPS) measurements were made using XPS measurements were carried out on K-Alpha spectrometer (Thermo Fisher Scientific, Waltham, MA, USA) using a monochromatic Al Kα radiation (1486.68 eV), with a spot size of 250 × 250 μm. A flood gun was used for charge compensation and the C1s line of 284.8 eV was used as a reference to correct the binding energies for charge energy shift.

## 3. Results and Discussion

**Figure 1:**
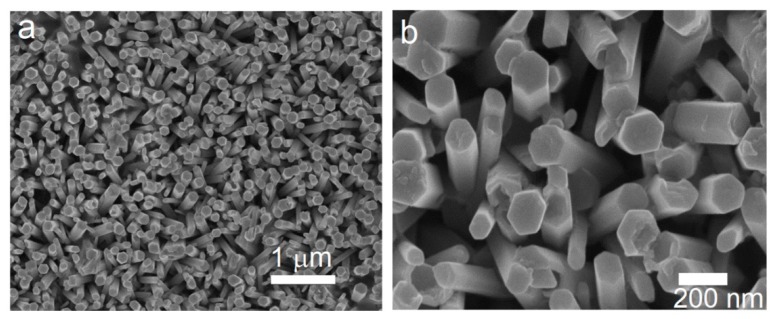
SEM microstructures of the as-prepared vertically aligned ZnO array (sample Z-nR) at (**a**) low magnification and (**b**) high magnification.

The SEM microstructures of the ZnO nanorods assembly at low and high magnification are presented in Figure 1. From the top view of the as-deposited sample, the nanorods appear to exhibit growth in a direction that is almost perpendicular to the substrate surface. The electron microscopy also revealed well-faceted hexagonal growth with an area density of ~15/µm^2^. The diameter and length of the nanorods was in the range of 100 to 150 nm and ~1 µm, respectively. The characteristic features of the nanorods assembly such as aspect ratio, area density, and degree of alignment depend on the crystalline quality of the seed as well as the processing conditions [38,39]. The faceted, hexagonal morphology becomes more evident at high magnification (Figure 1b). The deviation from ideal vertical growth of the ZnO nanorods and the not-so-perfect c-axis orientation (noticed in the XRD pattern) are caused by the polycrystalline nature of the ZnO seeds [40].

**Figure 2:**
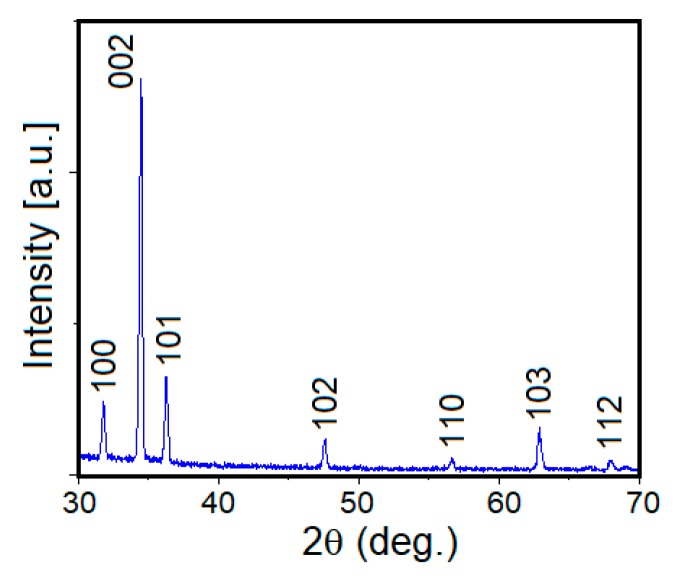
X-ray diffraction pattern of the as-made ZnO nanorods sample (Z-nR).

The X-ray diffraction pattern of the as-produced ZnO nanorods is presented in Figure 2. All the diffraction peaks were indexed to the ZnO hexagonal phase (space group: P63mc; JCPDS No. 36-1451). The most intense peak is located at 34.4° and can be assigned to the (002) crystallographic plane. From the presence of other peaks besides the preferred orientation, it can be deduced that the ZnO NR may either be polycrystalline with an overall preferred orientation or single crystals with most of them having the same preferred orientation [41]. The degree of vertical growth and the c-axis preferred orientation can be further enhanced through improvement in crystal quality of the ZnO seeds [42].

The values of the texture coefficient (*T_C_*) for the (100), (002), (101), and (103) diffraction peaks and the crystallite size (*D*) corresponding to the most intense (002) peak were calculated through Equations (1)–(3). The strain induced in the film due to lattice distortions, as determined using the Williamson–Hall method, is a function of line breadth (*β_hkl_*) and diffraction angle (*θ*) as given by Equation (4) [43,44,45],

(1) $$T_{c}\left(hkl\right)=\frac{I\left(hkl\right)/I_{0}\left(hkl\right)}{\frac{1}{n}\sum_{n}I\left(hkl\right)/I_{0}\left(hkl\right)}$$

(2) $$D=\frac{k\lambda }{\beta_{hkl}\;\cos\;\theta }$$

(3) $$\beta_{hkl}={\left[{\left(\beta_{hkl}\right)}^{2}{}_{measured}-{\left(\beta \right)}^{2}{}_{instrumental}\right]}^{1/2}$$

(4) $$\varepsilon =\frac{\beta_{hkl}}{4\;\tan\theta }$$

*I* and *I*_0_ are the measured relative intensities and the JCPDS standard intensities for the plane with *hkl* indices. On the other hand, *λ*, *k*, *β_hkl_*, *β_intrument_*, and *θ* are monochromatic wavelength (1.5405 Å), shape-factor (0.94), full-width-half-maximum (FWHM) of the diffraction peak with *hkl* planar indices, instrumental corrected integral breadth of the diffraction at 2*θ*, and the diffraction angle, respectively. The XRD data clearly demonstrate preferred crystallographic orientation along (002), as indicated by a higher value of 2.79 for *T_C_* (002) as compared to other diffraction peaks due its lowest surface free energy [46,47]. From the Scherrer Equation (2), the crystallite size was found to be 45.1 nm. Additionally, the annealing treatment induced high crystalline quality among the nanorods with strong (002) orientation. Assuming homogeneous isotropic nature of the ZnO crystals, the magnitude of strain in the annealed film was determined to be 1.45 × 10^−3^, which is quite low for the annealed ZnO film. It is noteworthy that XRD patterns and SEM microstructures of the ZnO NR specimens after plasma treatment were similar to those for the as-made ZnO NR (without plasma treatment). The XPS analysis, however, revealed that the surface chemistry of the nanorods was altered, as discussed later. 

**Figure 3:**
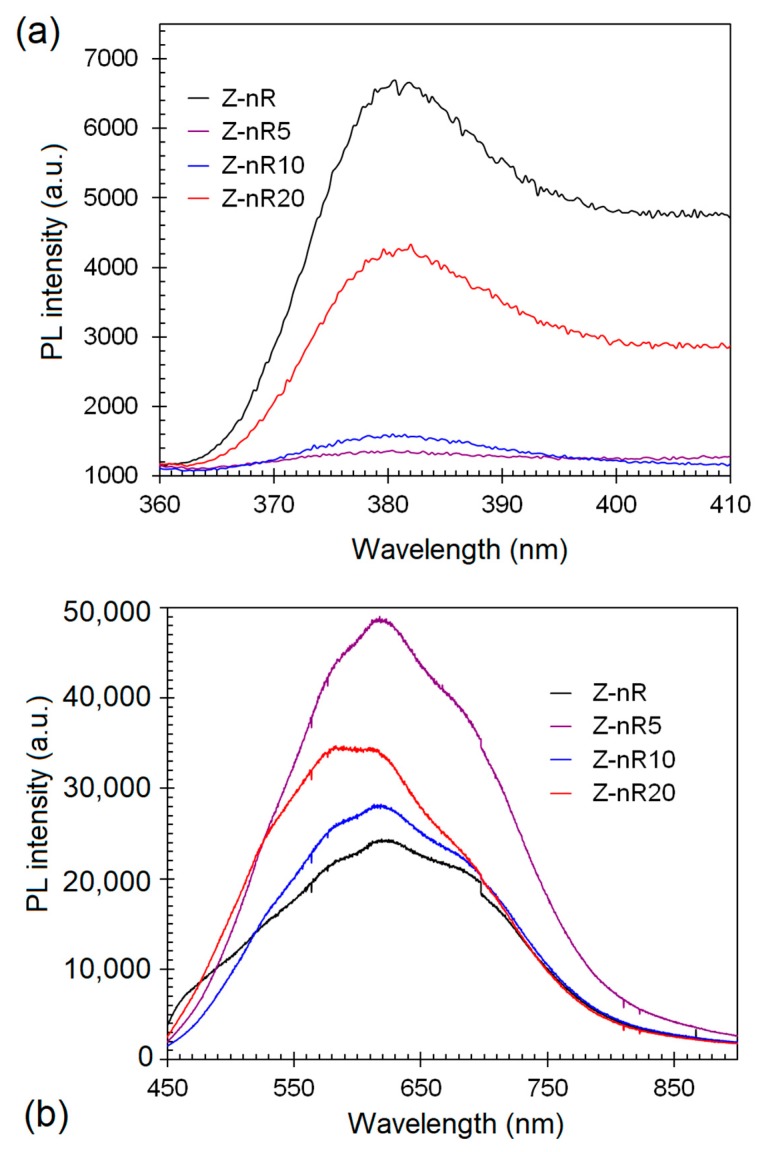
Photoluminescence spectra of the different Z-nR samples before and after SF_6_ plasma treatment in the (**a**) ultraviolet (UV) spectral regime and (**b**) visible spectrum.

The room temperature PL spectra of the ZnO NR samples before and after plasma treatment are shown in Figure 3. The band at 380 nm (Figure 3a) corresponds to 3.26 eV energy and is associated with the ZnO band gap emission [48]. The defect emissions due to structural defects in ZnO, such as *V_O_* and *Zn_i_*, are usually located in the spectral range of 450 to 650 nm [49]. Since the defect-related intensity is significantly higher than the band gap emission, the presence of crystallographic defects can be deduced in the as-deposited ZnO nanorods sample. It is noteworthy that the visible emission in all the samples was centered at 619 nm (2.0 eV) in the orange/red emission. The orange PL band with the maximum at 2.02–2.10 eV was earlier attributed either to oxygen interstitial atoms (*O_i_*) due to excess oxygen on the ZnO surface (2.02 eV) or to the hydroxyl group (OH) (2.09 eV) [50]. The orange emission is caused by transition from the Zn interstitial (*Zn_i_*) to the oxygen interstitial (*O_i_*) states. Such phenomena are generally observed in oxygen rich systems [51]. In the Z-nR sample (Figure 3b), the highest PL peak is centered at ~620 nm, which indicates that oxygen interstitials are the dominant defects even before plasma treatment. The treatment of the Z-nR with Ar/SF_6_ plasma led to a significant abatement of the intensity of the band gap emission in comparison with that of the Z-nR, particularly in case of Z-nR5 and Z-nR10 arrays. These reductions in band gap emission intensity may be ascribed to an increase in the defects density. These defects, in turn, trap the excited photons before the NBE recombination. In case of visible emission, the Z-nR5 and Z-nR20 samples demonstrate the maximum defect emission enhancement in the red/orange region. Thus, the degree of enhancement in the visible emission band for the three plasma-treated nanostructure arrays, in ascending order is Z-nR10 < Z-nR20 < Z-nR5. The *I_Vis_/I_UV_* ratio for the four samples, in descending order: Z-nR5 (36.4) > Z-nR10 (18.0) > Z-nR20 (8.16) > Z-nR (3.65). It seems that for the intermediate plasma treatment times of 5 and 10 min, the NBE peak is almost entirely quenched, followed by peak resurgence upon 20 min treatment, presumably due to generation of oxygen related defects. The effect of Ar/SF_6_ plasma treatment for ~20 min should be further investigated for shorter time intervals in order to establish any concrete surface chemistry–PL properties correlation. There are conflicting reports on the relative decrease, increase or no change in the relative intensities of the NBE (UV) or DLE (visible range) peaks, with the reported findings depending on several factors including ZnO morphology, plasma composition, plasma treatment time, that in turn, influence the defect types and concentration on ZnO surface besides attachment of hydroxyl groups [30,31,32,33,52,53]. 

There are many reports about quenching and suppression of green emission band in fluorine-doped ZnO (ZnO:F) films due to oxygen vacancies (*V_O_*) [52,53,54]. Upon F-doping, the *V_O_* defect density is decreased, causing a reduction in the green emission intensity since the F atoms effectively fill *V_O_* sites. In our case, an improvement in the orange/red emission of the visible spectral regime was noticed. Although the *V_O_* defects are responsible for the green emission (centered at ~500 nm) [49,50], the peak intensity is very weak in the as-made Z-nR sample and almost absent in the plasma-treated ZnO NR, as revealed by the PL spectra in Figure 3b. On the contrary, there are also reports on the appearance or band intensity enhancement of the red/orange emission from ZnO:F owing to an increased oxygen defect density [55] or replacement of oxygen by fluorine through the occupation of interstitial sites [56]. The orange/red emission enhancement upon plasma treatment of the ZnO nanorods samples, as noticed in our case, will be further discussed in the next section. 

**Figure 4:**
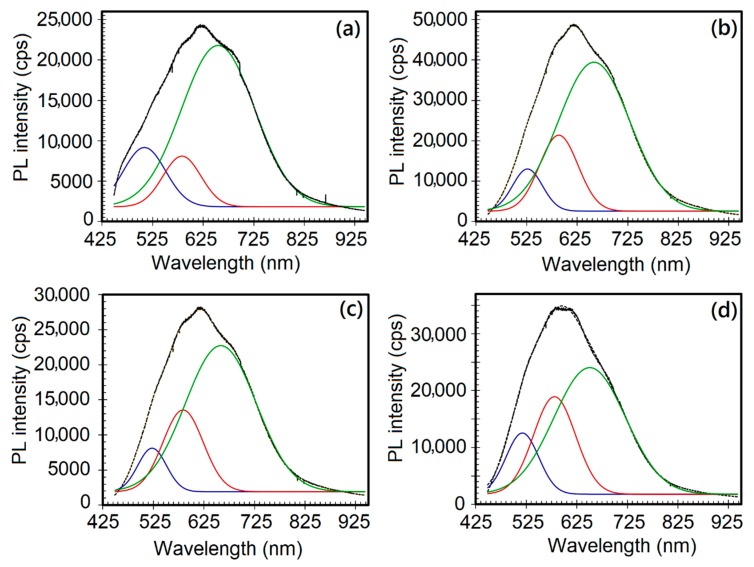
Deconvoluted PL spectra of the ZnO nanorods: (**a**) As-deposited nanorods (Z-nR), and after Ar/SF_6_ plasma treatment for (**b**) 5 min (Z-nR5), (**c**) 10 min (Z-nR10), and (**d**) 20 min (Z-nR20).

The PL spectra of the as-produced and plasma-treated ZnO nR samples were deconvoluted into component peaks, as demonstrated in Figure 4. In all the samples, the total PL visible emission had contributions from red, orange and green emissions. Although the red band intensity was much greater than those from orange or green bands, it is interesting to compare the relative spectral evolution of each band. Upon Ar/SF_6_ plasma treatment, a red shift in the green band position (2.44 eV) was observed with a maximum change by ~0.08 eV for 5 min treatment, in agreement with reports about annealed or O_2_ plasma-treated ZnO nanorods. On the other hand, there was no change in the respective positions of red (1.89 eV) and orange (2.13 eV) emission bands. A comparison of the red and orange band intensities with respect to that of green emission in terms of the *I_R_/I_G_* and *I_O_/I_G_* ratio for all the samples pointed towards a gradual decrease in the green emission intensity, presumably due to reduction in oxygen vacancies (*V_O_*) upon plasma treatment. The density of oxygen defects (*O_i_*), however, increased due to interaction between plasma species and the surface layers, thus giving rise to electron transitions responsible for red/orange emissions. The XPS data presented and discussed later in this section further confirm this observation.

**Figure 5:**
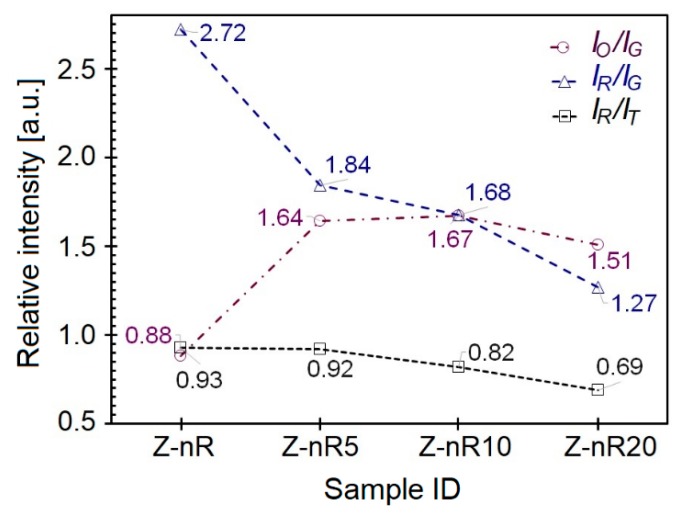
Change in orange (*I_O_*) and red (*I_R_*) emission intensities with respect to the green (*I_G_*) or total intensity (*I_T_*) from the PL spectra of the as-deposited and Ar/SF_6_ plasma-treated ZnO nanorods.

The change in intensities of the orange (*I_O_*), red (*I_R_*), and green (*I_G_*) emissions relative to the green or total spectral emissions, in terms of the *I_O_/I_G_*, *I_R_/I_G_*, and *I_R_/I_T_* ratios, are depicted for different ZnO nanorods samples in Figure 5. Upon Ar/SF_6_ plasma treatment, the intensity of red emission decreased from 2.72 (for the as-deposited sample) to the minimum value of 1.27 (for 20 min treatment) with an associated increase in the orange emission intensity. The sample Z-nR5 exhibited the greatest extent of reduction in the NBE intensity as well as the maximum enhancement in the broad emission in the visible range with the latter arising mainly from deep level emissions due to zinc vacancy related defects. It is understandable SF_6_ plasma generates SF_6_^+^ radicals that effectively passivate the ZnO surface through saturation of dangling bonds and diminishing the *V_O_* concentration [31]. 

**Figure 6:**
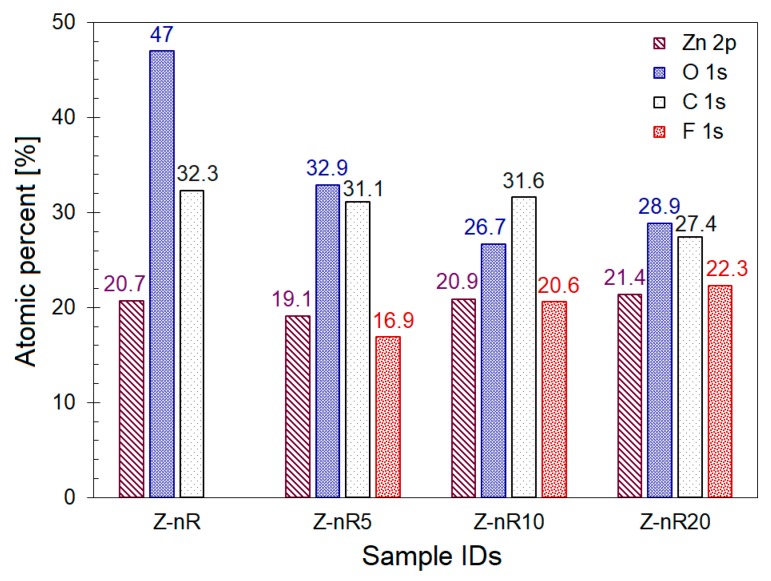
Bar chart showing atomic percentage of different elements as determined from the X-ray photoelectron spectroscopy (XPS) data.

In order to elucidate the PL emission behavior, XPS analyses were performed on the different ZnO nanorods samples before and after plasma treatment. The elemental composition (in atomic percent) of the different surfaces before and after plasma treatments are shown in Figure 6. While the oxygen content was found to decrease with increasing duration of the mixed Ar/SF_6_ plasma treatment, presumably due to incorporation of F at the surface region, the latter increases as the treatment time increases. There was no sulfur detected on any of the Z-nR5, Z-nR10, and Z-nR20 surfaces (after Ar/SF_6_ plasma treatment), implying that there are more F ions in the plasma (SF_6_) than S. Also, the reactivity of F ions is greater and stronger than that of S ions. The carbon element was also present in all the samples, it is attributed to the adsorbed carbon due to contamination.

**Figure 7:**
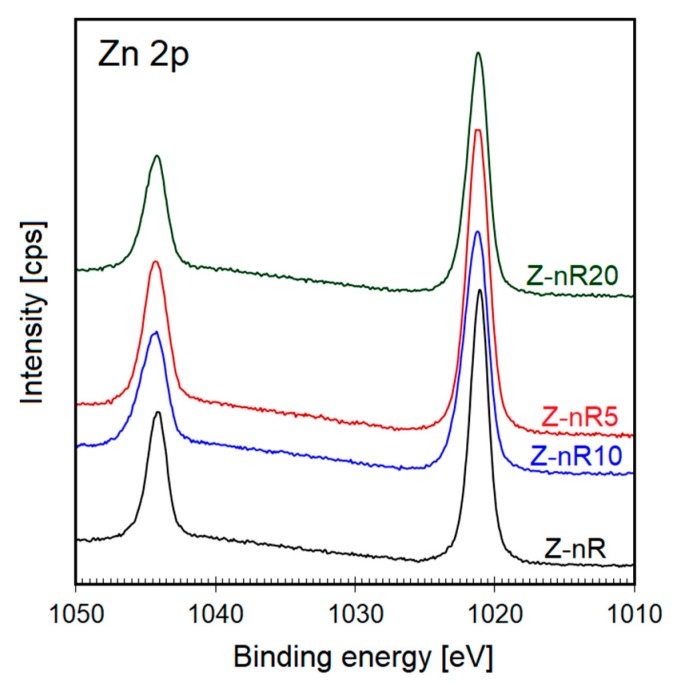
XPS Zn 2p core level spectra for the ZnO nanorods before and after Ar/SF_6_ plasma treatment.

The high-resolution XPS spectra of the as-deposited and plasma functionalized Z-nR samples of Zn 2p peaks are presented in Figure 7. The Zn 2p core lines of the Z-nR sample (no plasma treatment) comprise two distinct peaks that may be ascribed to the Zn 2p_3/2_ (1021.4 eV) and Zn 2p_1/2_ (1044.5 eV) with a spin–orbital splitting (Δ metal) of 23.1 eV. This is a direct confirmation of the Zn atoms to be in 2+ oxidation state [54]. From Figure 7, a slight shift of 0.2 V in the peak position towards higher binding energy values was noticed upon Ar/SF_6_ plasma treatment, possibly due to F^−^ ion incorporation into the ZnO lattice in the near-surface region thus causing the ZnO electronic band structure to be altered. Since electronegativity value for the F^−^ (3.98) is greater than that of O^2−^ (3.44), the net charge transfer from Zn (1.65) to F will be more dominant than that from Zn to O. Nevertheless, the value of Δ (metal) remained constant for all of the samples, indicating that the subatomic structure and the core-level of Zn in the ZnO host lattice is independent of the F from the plasma treatment [57,58].

**Figure 8:**
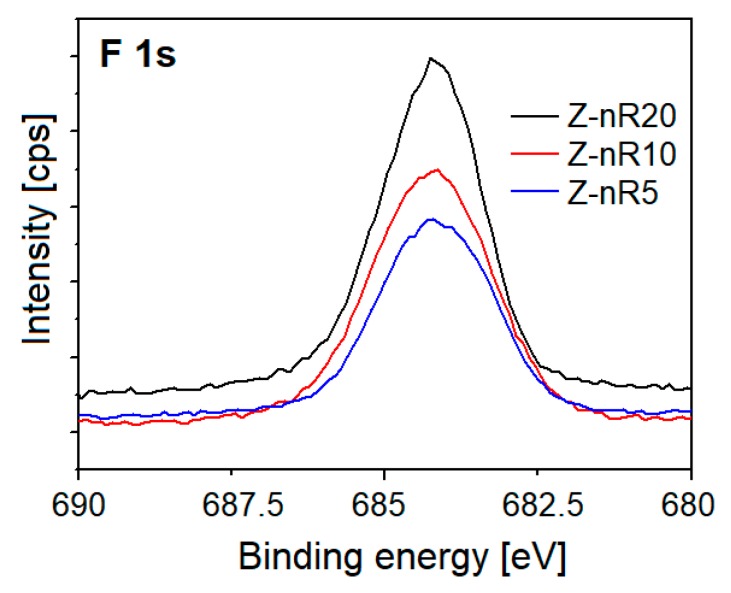
XPS F 1s core level spectra for the ZnO nanorods surface after Ar/SF_6_ plasma treatment.

The F^−^ incorporation into ZnO lattice can proceed in three possible ways: (i) formation of dative hydrogen bonds (OH–F) with surface hydroxyl groups, (ii) occupation of oxygen vacancies, and (iii) monosubstitution into oxygen lattice sites in the ZnO matrix. In order to quantify which one is dominant, the F 1s and O 1s XPS core level spectra were also carefully evaluated. The F 1s high-resolution spectra of the Z-nR samples at different plasma treatment times are shown in Figure 8. All the samples revealed the characteristic peak at 684.3 eV (F 1s peak) related to the F–Zn bond, which indicates replacement of the O by F upon fluorination, thus modifying the surface chemical composition of the ZnO. Upon SF_6_ plasma treatment for 5 and 10 min (Z-nR5 and Z-nR10), a slight shift of ~0.1–0.2 eV in the BE to higher values was observed, which may be associated with the presence of dangling bonds on the ZnO surface such as F–OH bonds with more F surrounded by oxygen in the ZnO.

**Figure 9:**
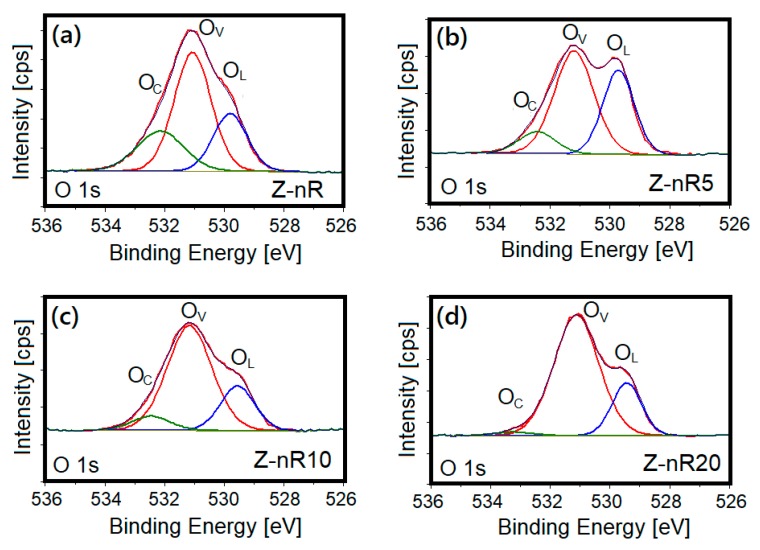
XPS O 1s deconvoluted core level spectra of (**a**) as-prepared ZnO NRs and ZnO NRs treated in Ar/SF_6_ plasma: (**b**) Z-nR5, (**c**) Z-nR10, and (**d**) Z-nR20.

The O 1s deconvoluted core level spectra of the Z-nR before and after SF_6_ plasma treatment are presented in Figure 9. The O 1s peak of ZnO is fitted by three nearly Gaussian components mainly centered at about 530, 531.5, and 532.5 eV with these peaks associated with O^2−^ species in the lattice (*O_L_*), oxygen vacancies or defects (*O_i_*), and chemisorbed or dissociated (*O_C_*) oxygen species such as hydroxyl group, H_2_O, O_2_, H, and –CO_3_, respectively [59,60,61]. During hydrothermal synthesis, oxygen adsorption from O_2_, CO_2_, H_2_O, etc. promotes appearance of the PL component peak for *O_C_*. The peak due to *O_L_* represents O^2−^ in the lattice structure or presence of Zn–O bonds. Additionally, the component peak characteristic of *O_i_* indicates generation of deep level point defects 0.7–1.0 eV below the bottom of conduction band with enhanced visible PL emission. The respective central positions for the three Gaussian components *O_L_*, *O_i_*, and *O_C_* that fit the O 1s peak and their percentages for the as-deposited and SF_6_ plasma-treated samples are listed in Table 1. It is evident from Table 1 that the relative fraction of the *O_C_* component decreases from approximately 23% to 2% with increasing plasma treatment time, implying greater extent of dissociation/conversion of the *O_C_* species initially present due to excess oxygen. Such intensity decrease in the *O_C_* component peak maybe attributed to the removal of loosely bound contaminants by the energetic species in low-pressure plasma. Also, fluorine may substitute oxygen vacancies (dangling zinc bonds) and/or oxygen atoms in the ZnO lattice, or even passivation of dangling oxygen bonds through formation of some intermolecular hydrogen bonds [30]. Comparison of the changes in the *O_C_* peak intensities with PL intensity evolution reveals that the PL intensity change is not linearly related with the *O_C_* surface content (by percent). A correlation between the PL intensity enhancement and the *O_C_* percentage on the nanostructures surface cannot be established. The presence of *O_i_* on the samples’ surfaces can be characterized by the equation, *O_i_* (%) = *O_i_*/(*O_i_* + *O_C_* + *O_L_*); this value was found to increase from ~1.26% for the as-prepared Z-nR to a maximum value of 3.2% for the Z-nR20 sample, probably due to fact that the Ar ions bombardment from the Ar/SF_6_ mixture causes generation of new oxygen defects. Among all the SF_6_ plasma-treated ZnO nanorods samples, the longer plasma treatment times of 10 and 20 min are considered to provide passivation to surface states and bulk defects along with maximum degree of F incorporation. This is in agreement with a recent study that reported formation of high-quality, highly-doped ZnO films upon flash lamp annealing in the millisecond range [62]. On the other hand, SF_6_ plasma treatment of the ZnO nanrorods (Z-nR5) for only 5 min induces a maximum enhancement in the ZnO defect band intensity, presumably due to more favorable surface conditions in terms of OH bond physisorption, oxygen incorporation into the ZnO lattice (*O_L_* peak in the XPS spectrum) and generation of F–OH and F–Zn bonds. 

This work demonstrates that plasma modification of the nanostructures and thin films can lead to fine tuning of the functional properties without causing any structural changes. When compared with other recent works on different nanoscale materials involving hybrid nanostructures or surface chemistry modification [63,64,65,66], novel functional properties can be carefully tuned for subsequent incorporation into microelectronic and/or optoelectronic devices.

## 4. Conclusions

ZnO nanorods arrays with high surface area and well-aligned crystallographic orientation were produced via hydrothermal method. The annealed arrays exhibit a crystallite size of 45 nm with (002) preferred orientation and a relatively low lattice strain. Upon argon/SF_6_ plasma treatment for up to 20 min, the PL intensity in the orange/red region of ZnO is enhanced by 2-fold compared to the ZnO sample without plasma treatment. Moreover, the PL intensity in the blue spectral regime is almost suppressed due to increased defect density. For 5 min Ar/SF_6_ plasma treatment, the PL broad band intensity maximizes due to maximum oxygen content at the film surface. This finding implies presence of hydroxyl group at the surface, more oxygen in the ZnO lattice (*O_L_*), fluorine incorporation in terms of F–Zn and F–OH bonds, and passivation of the surface states as well as bulk defects. These findings have implications for fundamental studies and industrial application of the plasma modified ZnO nanostructures in optoelectronic applications including visible light emitting devices and display systems. 

## Figures and Tables

**Table 1:** Deconvolution of the O 1s XPS peak in the different ZnO nanorods in terms of binding energy values and atomic percentages for the three O 1s component peaks.

Sample	O 1s Binding Energy (eV)	Percentage (%)
Z-nR	*O_L_*: 529.8	23.8
	*O_i_*: 531.0	55.1
	*O_C_*: 532.1	21.1
Z-nR5	*O_L_*: 529.7	35.1
	*O_i_*: 531.2	54.2
	*O_C_*: 532.4	10.7
Z-nR10	*O_L_*: 529.6	22.6
	*O_i_*: 531.2	69.3
	*O_C_*: 532.5	8.1
Z-nR20	*O_L_*: 529.5	21.7
	*O_i_*: 531.1	76.2
	*O_C_*: 533.4	2.1
